# Notice of Retraction and Replacement. Ryan and Markovitz. Estimated Savings From the Medicare Shared Savings Program. *JAMA Health Forum*. 2023;4(12):e234449

**DOI:** 10.1001/jamahealthforum.2024.0043

**Published:** 2024-04-05

**Authors:** Andrew M. Ryan

**Affiliations:** 1 Department of Health Services, Policy and Practice, Brown University School of Public Health, Providence, Rhode Island

**To the Editor** On behalf of my coauthor, I write to report errors that occurred in our Brief Report^1^ that was published on December 15, 2023, in *JAMA Health Forum*. In this study, using estimates from 2 studies and data on Medicare Shared Savings Program (MSSP) incentive payments for MSSP performance years 2013 to 2021, we found and reported that the “MSSP was associated with net losses to traditional Medicare, net savings to Medicare Advantage, and overall net savings to CMS.”

However, after publication of this study, I was contacted by a representative from the Medicare Payment Advisory Commission (MedPAC) who provided evidence that our calculations regarding the association between MSSP and MA benchmarks were incorrect. Apparently, MSSP incentive payments are incorporated into the US per capita cost (USPCC), which determines Medicare Advantage (MA) benchmarks. In the 2024 Medicare Rate Announcement released in 2023, a line item labeled “Innovation model shared savings (Includes APM)” is positive for Part A and Part B in all years, demonstrating how alternative payment model incentive payments (including those in the MSSP) increase the USPCC.^2^ In our published study, we had erroneously assumed that MSSP incentive payments were not incorporated into the calculation of the USPCC.

After correcting this error in our calculations, we found that the MSSP was associated with increases to MA benchmarks, totaling $191 million during the study period based on estimates from McWilliams et al^3^ (and $640 million based on estimates from MedPAC). Summing net losses from traditional Medicare and MA, we now find that the MSSP was associated with total losses to the US Centers for Medicare & Medicaid Services (CMS) of $775 million based on derived estimates from McWilliams et al^3^ and $2.063 billion based on MedPAC-derived estimates. This is different from our prior conclusion that the MSSP was associated with decreases to MA benchmarks and overall savings between $4.480 and $4.923 billion to CMS. Our finding that the MSSP was associated with net losses to traditional Medicare of between $584 million and $1.423 billion during the study period remains unchanged.

As a result of these errors and the corrections needed, we have requested that our original article be retracted and replaced. The replacement article includes corrections to our calculations of the estimated association between the MSSP and MA benchmarks in the Abstract and text. The key to Figure 2 has been corrected by replacing “Total Medicare” with “Total CMS.” Supplement 1 has also been corrected to include the revised methods, and a new eTable has been added that shows several of the values used to estimate the association between the net effect of the MSSP and benchmark payments to MA plans.

We regret these errors and apologize to the readers and editors for any confusion. We appreciate the opportunity to publish the corrected article and confirm that there are no other errors. With this corrected version, a copy of the original article with the errors highlighted and a copy with the corrections highlighted are included as new Supplements 3 and 4, respectively.
